# Supplementary material for: The causal relationship between the human gut microbiota and pyogenic arthritis: a Mendelian randomization study
Source: Front Cell Infect Microbiol. 2024 Nov 26;14:1452480. doi: 10.3389/fcimb.2024.1452480 (PMC11629706; doi:10.3389/fcimb.2024.1452480)
Supplement: Supplementary file 1 [file DataSheet1.zip › mendelian test/ebi-a-GCST90027650.csv_forest.pdf]

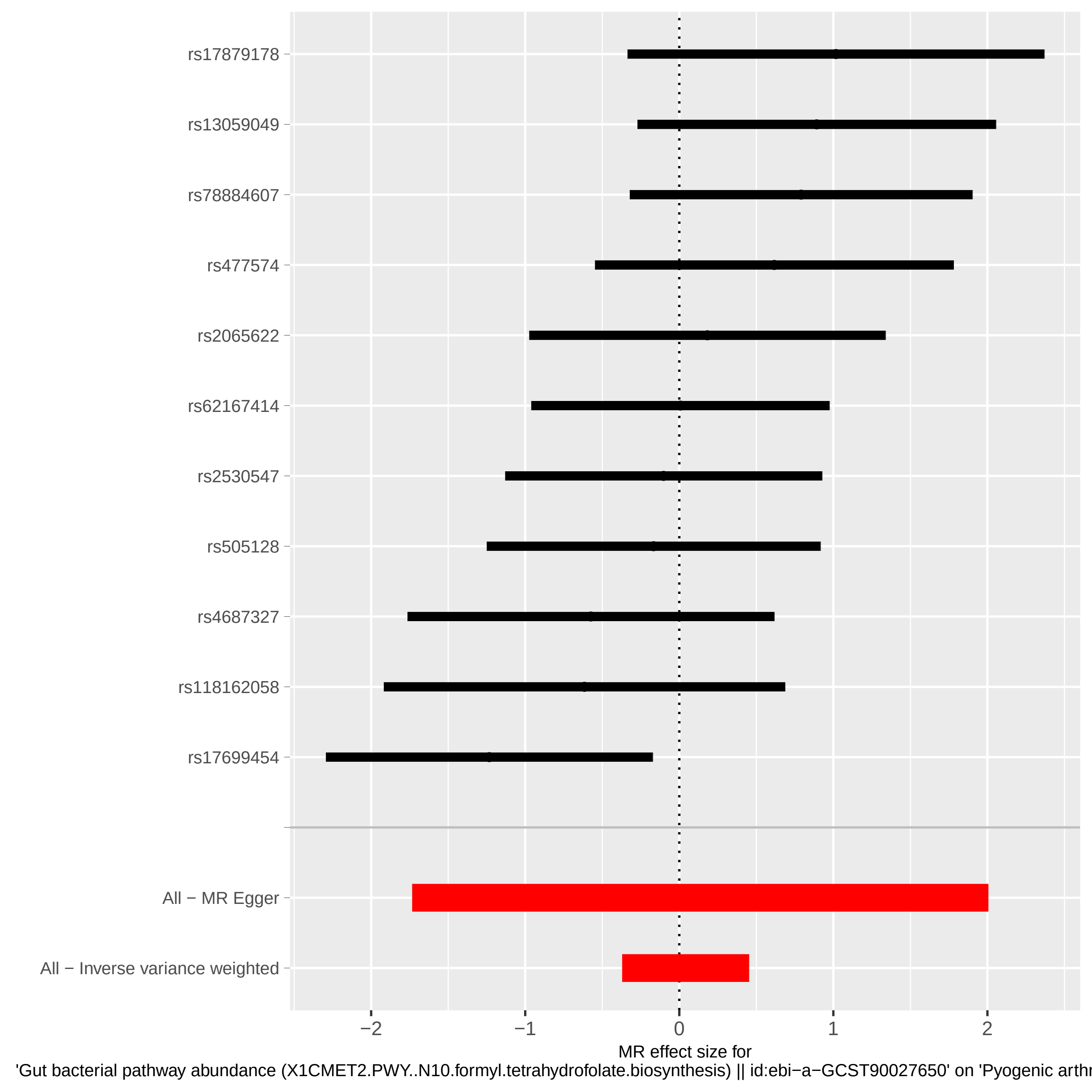
rs17879178
rs13059049
rs78884607
rs477574
rs2065622
rs62167414
rs2530547
rs505128
rs4687327
rs118162058
rs17699454
All − MR Egger
All − Inverse variance weighted
−2
−1
0
1
2
MR effect size for
'Gut bacterial pathway abundance (X1CMET2.PWY..N10.formyl.tetrahydrofolate.biosynthesis) || id:ebi−a−GCST90027650' on 'Pyogenic arth
